# Rationale and design of the prevention of paclitaxel-related neurological side effects with lithium trial – Protocol of a multicenter, randomized, double-blind, placebo- controlled proof-of-concept phase-2 clinical trial

**DOI:** 10.3389/fmed.2022.967964

**Published:** 2022-08-11

**Authors:** Petra Huehnchen, Nikola Bangemann, Sandra Lischewski, Stefanie Märschenz, Friedemann Paul, Tanja Schmitz-Hübsch, Jens-Uwe Blohmer, Cornelia Eberhardt, Geraldine Rauch, Agnes Flöel, Sophie Adam, Philipp Schwenkenbecher, Ivo Meinhold-Heerlein, Oliver Hoffmann, Tjalf Ziemssen, Matthias Endres, Wolfgang Boehmerle

**Affiliations:** ^1^Charité – Universitätsmedizin Berlin, Corporate Member of Freie Universität Berlin, Klinik und Hochschulambulanz für Neurologie, Humboldt-Universität zu Berlin, Berlin, Germany; ^2^Berlin Institute of Health at Charité – Universitätsmedizin Berlin, Berlin, Germany; ^3^Carl-Thiem-Klinikum Cottbus, Klinik für Senologie und Systemische Gynäkoonkologie mit Brustzentrum, Cottbus, Germany; ^4^Charité – Universitätsmedizin Berlin, Corporate Member of Freie Universität Berlin, Humboldt-Universität zu Berlin, NeuroCure Clinical Research Center (NCRC), Berlin, Germany; ^5^Experimental and Clinical Research Center, A Cooperation Between the Max Delbrück Center for Molecular Medicine in the Helmholtz Association, Charité – Universitätsmedizin Berlin, Berlin, Germany; ^6^Charité – Universitätsmedizin Berlin, Corporate Member of Freie Universität Berlin, Humboldt-Universität zu Berlin, Experimental and Clinical Research Center, Berlin, Germany; ^7^Max Delbrück Center for Molecular Medicine in the Helmholtz Association (MDC), Berlin, Germany; ^8^Charité – Universitätsmedizin Berlin, Corporate Member of Freie Universität Berlin, Humboldt-Universität zu Berlin, Klinik für Gynäkologie und Brustzentrum, Berlin, Germany; ^9^Charité – Universitätsmedizin Berlin, Corporate Member of Freie Universität Berlin, Humboldt-Universität zu Berlin, Department of Pharmacy, Berlin, Germany; ^10^Charité – Universitätsmedizin Berlin, Corporate Member of Freie Universität Berlin, Humboldt-Universität zu Berlin, Institut für Biometrie und Klinische Epidemiologie, Berlin, Germany; ^11^Universitätsmedizin Greifswald, Department of Neurology, Greifswald, Germany; ^12^German Center for Neurodegenerative Diseases (DZNE), Greifswald, Germany; ^13^Mammazentrum Hamburg, Hamburg, Germany; ^14^Medizinische Hochschule Hannover, Department of Neurology, Hanover, Germany; ^15^Universitätsklinikum Giessen, Klinik für Gynäkologie und Geburtshilfe, Giessen, Germany; ^16^Universitätsklinikum Essen, Klinik für Frauenheilkunde und Geburtshilfe, Essen, Germany; ^17^Universitätsklinikum Carl Gustav Carus, Klinik und Poliklinik für Neurologie, Dresden, Germany; ^18^Charité – Universitätsmedizin Berlin, Corporate Member of Freie Universität Berlin, Humboldt-Universität zu Berlin, Center for Stroke Research Berlin (CSB), Berlin, Germany; ^19^German Center for Neurodegenerative Diseases (DZNE), Berlin, Germany; ^20^German Centre for Cardiovascular Research (DZHK), Partner Site Berlin, Berlin, Germany

**Keywords:** chemotherapy, paclitaxel, lithium, polyneuropathy, cognitive impairment, neurotoxicity, prevention

## Abstract

**Introduction:**

Chemotherapy-induced polyneuropathy (CIPN) and post-chemotherapy cognitive impairment (PCCI) are frequent side effects of paclitaxel treatment. CIPN/PCCI are potentially irreversible, reduce quality of life and often lead to treatment limitations, which affect patients’ outcome. We previously demonstrated that paclitaxel enhances an interaction of the Neuronal calcium sensor-1 protein (NCS-1) with the Inositol-1,4,5-trisphosphate receptor (InsP_3_R), which disrupts calcium homeostasis and triggers neuronal cell death via the calcium-dependent protease calpain in dorsal root ganglia neurons and neuronal precursor cells. Prophylactic treatment of rodents with lithium inhibits the NCS1-InsP_3_R interaction and ameliorates paclitaxel-induced polyneuropathy and cognitive impairment, which is in part supported by limited retrospective clinical data in patients treated with lithium carbonate at the time of chemotherapy. Currently no data are available from a prospective clinical trial to demonstrate its efficacy.

**Methods and analysis:**

The PREPARE study will be conducted as a multicenter, randomized, double-blind, placebo-controlled phase-2 trial with parallel group design. *N* = 84 patients with breast cancer will be randomized 1:1 to either lithium carbonate treatment (targeted serum concentration 0.5–0.8 mmol/l) or placebo with sham dose adjustments as add-on to (nab-) paclitaxel. The primary endpoint is the validated Total Neuropathy Score reduced (TNSr) at 2 weeks after the last (nab-) paclitaxel infusion. The aim is to show that the lithium carbonate group is superior to the placebo group, meaning that the mean TNSr after (nab-) paclitaxel is lower in the lithium carbonate group than in the placebo group. Secondary endpoints include: (1) severity of CIPN, (2) amount and dose of pain medication, (3) cumulative dose of (nab-) paclitaxel, (4) patient-reported symptoms of CIPN, quality of life and symptoms of anxiety and depression, (5) severity of cognitive impairment, (6) hippocampal volume and changes in structural/functional connectivity and (7) serum Neurofilament light chain protein concentrations.

**Ethics and dissemination:**

The study protocol was approved by the Berlin ethics committee (reference: 21/232 – IV E 10) and the respective federal agency (Bundesinstitut für Arzneimittel und Medizinprodukte, reference: 61-3910-4044771). The results of the study will be published in peer-reviewed medical journals as well as presented at relevant (inter)national conferences.

**Clinical trial registration:**

[https://www.drks.de/drks_web/navigate.do?navigationId=trial.HTML&TRIAL_ID=DRKS00027165], identifier [DRKS00027165].

## Introduction

### The medical problem

Neurotoxic sequelae are among the most common side effects of cytotoxic chemotherapy and affect a large number of patients. The development of chemotherapy-induced polyneuropathy (CIPN) is a well-recognized albeit pathophysiologically incompletely understood side effect in the peripheral nervous system. With a prevalence of 0.5% in the general population, CIPN is more prevalent than Multiple Sclerosis or Parkinson’s disease (1). Additionally, up to 50% of patients report a decline of cognitive function in temporal correlation to systemic chemotherapy, which has been termed post-chemotherapy cognitive impairment (PCCI) [reviewed by (2)]. To date there are no options to prevent or treat CIPN or PCCI. A recent retrospective study from the Department of Neurology at the Mayo Clinic demonstrated that 53% of all patients receiving a neurotoxic chemotherapy had clinical records to allow the diagnosis of CIPN, a figure that still likely underestimates the incidence of this condition. The burden of disease in this patient cohort was highly relevant and the condition often persisted in the follow-up period of 5 years (3). Patients suffering from neuropathic pain, a frequent observation in CIPN, experience substantially lower health-related quality of life to an extent comparable to conditions like stroke or severe heart failure (4). Another study in breast cancer survivors with CIPN due to a taxane-based chemotherapy showed a significant burden of disease with more frequent falls as well as higher rates of anxiety, depression and insomnia (5). In summary, neurological sequelae present a major burden of disease for cancer survivors following a neurotoxic chemotherapy. However, neurotoxicity not only increases the burden of disease, it also affects cancer prognosis by necessitating treatment changes. Despite this high relevance for patients, little research efforts let alone therapeutic strategies are allocated to neurological side effects of cytotoxic drugs. Given the medical need, the development of a targeted neuroprotective therapy for CIPN/PCCI is evident.

In the past, the development of neuroprotective strategies from “bench to bedside” has failed in a number of neurological diseases including stroke and Parkinson’s disease and has even been termed “translational roadblock.” One possible explanation for this failure is the unpredictable onset of neuronal cell death and damage as well as the plethora of pathophysiological mechanisms leading to neurodegeneration. In contrast, the well-defined and known time point of chemotherapy-induced neurotoxicity presents a major conceptual advantage. Despite these advantages, a number of prevention and therapy studies in CIPN patients have been negative and symptomatic therapies originally established for other medical conditions are often insufficient (6). Several reasons may account for past failures of preventive co-medications: First, the variability of the disease course makes the choice of study endpoints challenging. CIPN can lead to both a gain of function, i.e., positive or “plus” symptoms like pain, par- and dysesthesia, but also loss of function, i.e., negative or “minus” symptoms such as numbness, which leads to fine motor impairment and ataxia. Many studies therefore focused only on a few aspects of CIPN such as neuropathic pain, which meant that other important features of the disease may have been overlooked. This observation has since triggered the development of more robust assessment tools such as the (reduced) Total Neuropathy Score (TNSr) (7, 8). Second, past studies often suffered from considerable heterogeneity of the cytotoxic drugs and patient cohorts with different tumor entities. This heterogeneity may have diluted potential treatment effects. These observations led to the formulation of recommendations for future studies (9), which were incorporated in the present protocol.

### Evidence

The trial outlined here investigates neurotoxicity caused by (nab-) paclitaxel (PTX) in women with breast cancer. In 57–82% of treated patients (nab-) paclitaxel causes neurotoxic side effects in the peripheral and central nervous system (10). (Nab-) paclitaxel works by stabilizing the microtubule network, thus inhibiting the disassembly of the spindle apparatus during mitosis and has become widely used in the treatment of many solid tumors (e.g., breast and ovarian cancer, non-small cell lung cancer, head and neck tumors, etc.). Patients receiving a cumulative dose of (nab-) paclitaxel >300 mg/m^2^ body surface frequently develop a sensory axonal polyneuropathy (11, 12), which often presents with paresthesia, allodynia, and neuropathic pain making it particularly difficult to treat. While hyper-stabilization of microtubules with subsequent damage of the axonal cytoskeleton and inhibition of axonal transport were observed in paclitaxel-treated neurons at dosages from 10 to 200 μM (13, 14), cell death of dorsal root ganglia neurons already occurs at much smaller dosages around 100 nM, suggesting that additional mechanisms other than effects merely mediated through paclitaxel’s microtubule interference contribute to paclitaxel-induced polyneuropathy. Indeed, the molecular mechanism by which paclitaxel causes cell death in dorsal root ganglia neurons *in vitro* involves an enhanced interaction of the neuronal calcium sensor-1 (NCS-1) protein with the inositol-1,4,5-trisphosphate-receptor (InsP_3_R), which results in a calcium (Ca^2+^) efflux from the endoplasmic reticulum into the cytosol (15). Increased Ca^2+^ concentrations trigger the Ca^2+^ dependent protease calpain, which activates caspase-dependent apoptosis in sensory neurons and neural stem cells and leads to a cleavage of NCS-1 by calpain (16, 17). An additional InsP_3_R-tubulin interaction as a significant contributor to paclitaxel-induced cell death in sensory neurons could not be identified as no association of these proteins was found in co-immunoprecipitation experiments (15). We then demonstrated that lithium ions inhibit the paclitaxel-enhanced interaction of NCS-1 with the InsP_3_R (18). Additionally, a pre-treatment of human induced pluripotent stem cell-derived sensory neurons (hiPSC-DSN) with therapeutic lithium concentrations was able to ameliorate paclitaxel-induced apoptosis (19). Preclinical studies in paclitaxel-treated rodents further demonstrated that preventive lithium carbonate therapy also ameliorates the development of paclitaxel-induced polyneuropathy and post-chemotherapy cognitive impairment without evidence for an altered cytotoxic activity in malignant cells, respectively, tumor xenograft animal models (16, 20, 21). To date only indirect evidence exists, that such a co-medication with lithium carbonate is also effective in humans: Ehrlich and co-workers published preliminary results from a retrospective chart review, which concluded that treatment with lithium carbonate or valproic acid for other indications at the time of neurotoxic chemotherapy was associated with a decreased incidence of CIPN (22). Additional evidence highlighting lithium’s neuroprotective effects also speak in favor of a proof-of-concept clinical trial with a lithium carbonate co-medication to prevent paclitaxel-induced polyneuropathy: besides the proposed NCS-1/InsP_3_R mechanism, lithium has immunomodulatory properties by inhibiting inflammatory-associated pathways such as glycogen synthase kinase-3 beta (GSK-3ß), nuclear factor ‘kappa-light-chain-enhancer’ of activated B-cells (NF-κB) and signal transduction and activator of transcription (STAT) and thereby reducing inflammatory cytokines such as interleukin-6 (IL-6) (23). Particularly NF-κB translocation and subsequent IL-6 production by sensory neurons are major contributors to paclitaxel-induced polyneuropathy (24). Additionally, lithium is a known inhibitor of toll-like receptor 4 (TLR-4) expression (25), which is responsible for a secondary macrophage invasion of dorsal root ganglia neurons observed after paclitaxel treatment in rodents (26). Pretreatment with lithium also decreases Ca^2+^ currents through the transient receptor potential cation channel subfamily V member 4 (TRPV4), which are elevated in the presence of paclitaxel (27, 28). Furthermore, lithium increases the expression of brain-derived neurotrophic factor (BDNF) as well as genes associated with neuroprotection such as Bcl2 and Bcl-XL and decreases the expression of pro-apoptotic genes such as Bax, Bad, and caspases-3 (29), many of which are also known to be key players in paclitaxel-induced neurodegeneration (30).

In summary, our trial hypothesis is supported by data obtained by different groups in different cell and animal models as well as by retrospective clinical data. Based on these findings, we developed a protocol for a multicenter, randomized, double-blind, placebo-controlled proof-of-concept phase-2 trial in a clearly defined cohort of breast cancer patients, who are about to be treated with (bi-) weekly (nab-) paclitaxel infusions, to demonstrate that this approach is efficacious and safe.

### The need for a prospective clinical trial

Even though a beneficial effect of lithium carbonate in preventing neurotoxicity of cytotoxic drugs other than paclitaxel remains to be investigated, the preclinical and clinical evidence warrants this investigator-initiated trial (IIT). Lithium carbonate is a readily available and well-established drug presently used in the treatment of different psychiatric conditions with target steady-state serum concentrations of 0.5–1.2 mmol/l. It is also one of the most thoroughly characterized medications on the market (31). Current medical practice in case of CIPN development includes the reduction or termination of the chemotherapeutic drug, which directly affects patient outcome. Additional symptomatic treatment strategies for CIPN involve pain medication, which is frequently unsatisfactory to both patients and physicians alike. No pharmacological treatment exists for cognitive deficits following chemotherapy. If proven successful, the proposed approach would not only target both central as well as peripheral nervous system side effects of (nab-) paclitaxel chemotherapy, but furthermore implement a significant guideline change.

## Methods and analysis

### Study aims and hypothesis

#### Primary

The main objective of this proof-of-concept phase-2 clinical trial is to test the hypothesis, that a co-medication with oral lithium carbonate (Quilonum^®^ retard), adjusted to target lithium serum levels of 0.5–0.8 mmol/l, is able to reduce the burden of chemotherapy-induced polyneuropathy (CIPN) compared to placebo treatment in breast cancer patients undergoing neurotoxic chemotherapy with weekly or biweekly (nab-) paclitaxel infusions.

#### Secondary

The goal is to examine whether:

• A co-medication with lithium carbonate results in a reduced number of patients with moderate to severe CIPN,
• A co-medication with lithium carbonate leads to less intake or lower doses of pain medication.
• Patients with the co-medication lithium carbonate tolerate higher cumulative doses of (nab-) paclitaxel,
• A co-medication with lithium carbonate leads to fewer and less severe self-reported symptoms of CIPN,
• A co-medication with lithium carbonate improves patients’ quality of life,
• A co-medication with lithium carbonate reduces post-chemotherapy cognitive impairment caused by (nab-) paclitaxel,
• A co-medication with lithium carbonate ameliorates the decrease in hippocampal volume and changes in structural and functional connectivity,
• A co-medication with lithium carbonate leads to lower concentrations of neurofilament light chain protein in serum (NFL_*s*_).

### Study design and sample

**FIGURE 1:**
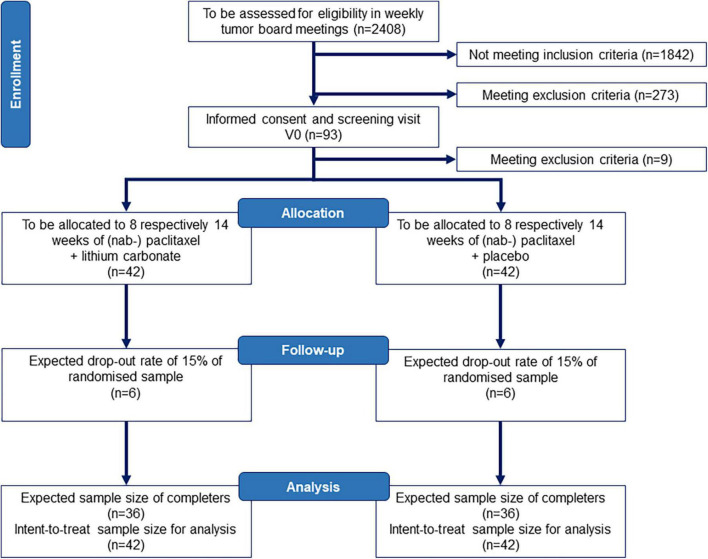
Study flow chart according to CONSORT. We assume that we need to screen *n* = 2408 patients with breast cancer according to punch biopsy or surgical resection pathology for eligibility at five to eight recruiting centers to enroll *n* = 93 and randomize *n* = 84 patients aged 18–70 years. The sample size and drop-out rate (15%) is conservatively based on other studies in CIPN (34, 35) and data from our own pilot study of CIPN development in breast cancer patients (36) as well as treatment effects for lithium carbonate in the prevention of CIPN in rodents (16, 21). CONSORT, Consolidated Standards of Reporting Trials.

The study protocol has been prepared in accordance with the Standard Protocol Items: Recommendations for Interventional Trials (SPIRIT) 2013 statement (32, 33). Please see Supplementary Table 1 for the SPIRIT checklist. The PREPARE trial is an investigator initiated study, which evaluates a co-medication with oral lithium carbonate medication to prevent (nab-) paclitaxel-induced neurotoxicity. The study will enroll *n* = 93 patients recruited from five to eight study sites in Germany. A total of *n* = 84 patients will be randomized to one of two groups: Group 1 will receive lithium carbonate with lithium serum levels titrated to 0.5–0.8 mmol/l add-on to (nab-) paclitaxel chemotherapy, whereas Group 2 will receive an identical looking placebo and undergo dose adjustments according to sham lithium serum concentrations add-on to (nab-) paclitaxel. Investigators, study physicians, study nurses, primary treating physicians (gynecologists and oncologists) and patients will be blinded as to the treatment. Two physicians from the Dept. of Psychiatry at Charité – Universitätsmedizin Berlin, who are highly experienced in lithium carbonate therapy and otherwise not involved in the trial or care of these patients, will assess the lithium serum levels for all patients and create sham lithium serum levels for all placebo patients. Figure 1 presents an overview of the study design. The first patient in is expected in June 2022 and last patient out in November 2024.

### Sample size calculation

This is a proof-of-concept trial, as the current level of evidence is limited. Therefore, the considerations underlying the sample size calculation correspond to a higher level of uncertainty than in a classical confirmatory trial. Sample size calculation is based on the primary endpoint TNSr at 2 weeks after the last (nab-) paclitaxel infusion (study visit V12 for biweekly [q2w] paclitaxel or V16 for weekly [q1w] paclitaxel infusions) adjusted for the baseline value in the intention-to-treat population including all randomized patients. The aim is to demonstrate, that the lithium carbonate group is superior to the placebo control group, i.e., that the mean TNSr 2 weeks after the last paclitaxel infusion (V12 or V16, respectively), adjusted for baseline, is lower in the lithium carbonate group than in the placebo control group. Sample size calculations are be based on a two-sample-*t*-test. As the ANCOVA model adjusting for the baseline scores, which is used for the primary efficacy analysis, will yield a power advantage compared to the two-sample *t*-test, which ignores the influence of different baseline values, this strategy for sample size calculation defines a conservative approach. Published TNSr values following paclitaxel therapy report mean TNSr values between 11.1 and 11.9 points after chemotherapy with a standard deviation of 3.4–3.6, however, based on low sample sizes (34). Another study reported pre-chemotherapy mean TNSr values of 1.6 and post-chemotherapy values of 5.6 (35), which is in line with data from our own pilot study (CICARO, NCT02753036) including 23 breast cancer patients (36). Here, we observed a mean TNSr value increase from 1.6 at baseline to 5.0 at the follow-up time point (SD 3.5). The existing evidence is summarized in Table 1. These data are used to provide estimates of the TNSr value development in the placebo control group (placebo add-on standard medication with [nab-] paclitaxel). Currently, no published data exists to assess the effect of a lithium carbonate therapy on prevention of CIPN. Therefore, the treatment effect is estimated based on our preclinical data (16). As the study is randomized, we assume that both groups start from the same TNSr baseline value of 1.6 score points. In the placebo arm, we assume that the mean TNSr value at the follow-up visit will increase to 5.0 score points with a standard deviation of 3.5. We estimate the treatment effect to be 50% reduced compared to the mean TNSr score in the placebo control group (16, 37), i.e., a maximum increase of the mean TNSr value to 2.5 score points at the follow-up time-point in the lithium carbonate group with an equal standard deviation of 3.5. That leads to a standardized effect size between groups at the follow-up time point of (5.0–2.5)/(3.5) ≈ 0.71. We conservatively calculated with an effect size of 0.6. The required sample size to find a significant effect with a power of 0.8 at a two-sided significance level of 0.1 is given by 72 patients (36 per treatment arm, calculated with nQuery 8.4.1). A significance level of 0.1 is an accepted threshold for proof-of-concept trials.

**TABLE 1:** Existing evidence for the placebo arm used for sample size calculation.

Pre chemotherapy Mean (*SD*)	Post chemotherapy Mean (*SD*)	References
	11.1–11.9 (3.4–3.6)	(34)
1.6 (–)	5.6 (–)	(35)
1.6 (2.0)	5.0 (3.5)	Own pilot data: NCT02753036 (36)

Regarding the primary endpoint, possible intercurrent events according to ICH E9 addendum are defined by a discontinuation of the trial medication lithium carbonate/placebo. Possible reason for a discontinuation of lithium carbonate/placebo may be:

• The patient meets one or more of the discontinuation criteria.
• The patient is repeatedly incompliant regarding the treatment with lithium carbonate/placebo.
• The patient wishes to stop the lithium carbonate/placebo treatment.
• The patient has to abandon chemotherapy treatment with (nab-) paclitaxel.

All collected data will be included in the analyses also in case of intercurrent events. Exclusion of data where an intercurrent event has occurred is not intended. Therefore, the primary estimand targets the clinical question “Does lithium carbonate prevent an increase in TNSr vs. placebo regardless of whether the trial medication was discontinued?” Given the exploratory status of the PREPARE trial and the study collective of cancer patients, we conservatively calculated a maximum of 15% of patients lost to follow-up with regards to the assessment of the primary endpoint. To account for these 15% drop-out, we plan to randomize *n* = 84 patients. As up to 10% of patients may be screening failures due to preexisting neuropathy (TNSr > 5 points, see exclusion criteria), the number of patients to be recruited is *n* = 93.

### Eligibility criteria and assessment

The key inclusion criteria for the PREPARE trial are as follows: (i) written informed consent; (ii) the patient has the capacity to give consent (she is able to understand the nature and anticipated effects/side effects of the proposed medical intervention); (iii) age 18 to 70 years; (iv) recent diagnosis of breast cancer to be treated with weekly (q1w) or biweekly (q2w) (nab-) paclitaxel infusions; (v) Karnofsky Index ≥ 70%.

Patients may be excluded from participation if one or more of the following exclusion criteria are fullfilled:

– The patient is pregnant or breastfeeding.
– The patient with childbearing potential is unwilling to use an acceptable form of contraception.
– The patient did or does participate in other interventional trials (6 months before and at the time of this trial).
– The patient is legally detained in an official institution.
– The patient is an employee of the investigator study site, or a family member of the employees or the investigators, or otherwise dependent on the sponsor, the investigators or study physicians.
– The patient has previously received neurotoxic chemotherapy (especially taxanes, platinum compounds, vinca alkaloids, and proteasome inhibitors).
– The patient has a pre-existing polyneuropathy with a baseline TNSr of >5 points.
– The patient has a history of current or former alcohol or drug abuse or a carbohydrate deficient transferrin (CDT) of >2.4% or a positive urine drug screening for amphetamine, barbiturates, benzodiazepines, cocaine, methamphetamine, methadone, opiates, tetrahydrocannabinol, that cannot be explained by prescribed medication.
– The patient has a cardiac pacemaker or other non-MRI suitable implants.
– Diagnosis of current COVID-19 illness.
– Lithium carbonate:
  ∘ The patient has a known allergy against lithium carbonate or other compounds of the investigational medical product (IMP).
  ∘ The patient has one of the following confirmed diagnoses: Brugada-Syndrome, myasthenia gravis, M. Addison, myeloid leukemia, psoriasis, epilepsy, severe cardiac arrhythmia, severe hyponatremia, severe dehydration, severe hypothyroidism, acute myocardial infarction, acute kidney failure, hereditary galactose intolerance, lactase insufficiency or glucose-galactose-malabsorption.
  ∘ The patient’s family history is positive for confirmed lethal sudden cardiac arrest.
  ∘ The patient uses phenytoin, fosphenytoin, carbamazepine, methyldopa, tricyclic antidepressants.
  ∘ The patient has chronic renal disease with a eGFR <60 ml/min/1.73 m^2^
– Placebo:
  ∘ The patient has a known allergy against cellulose or lactose.
– (Nab-) paclitaxel:
  ∘ The patient has a known allergy against (nab-) paclitaxel or other components of the standard-medical product (SMP).
  ∘ The patient has a diagnosis of severe liver failure.

### Randomization and blinding

Patients that consented to participation and are still eligible after the screening visit V0 are thereafter randomized 1:1 to lithium carbonate or placebo add-on to (nab-) paclitaxel chemotherapy (LI + PTX vs. PL + PTX) based on a randomization list provided by the trial statistician. The randomization list is generated in nQuery v8.4.1 using block randomization of variable block length stratified by study site. Only the trial statistician and the pharmacy at Charité – Universitätsmedizin Berlin are in possession of the randomization lists. Investigators and all clinical personnel conducting the trial will be blinded toward the block lengths to avoid that the group application is predictable.

**FIGURE 2:**
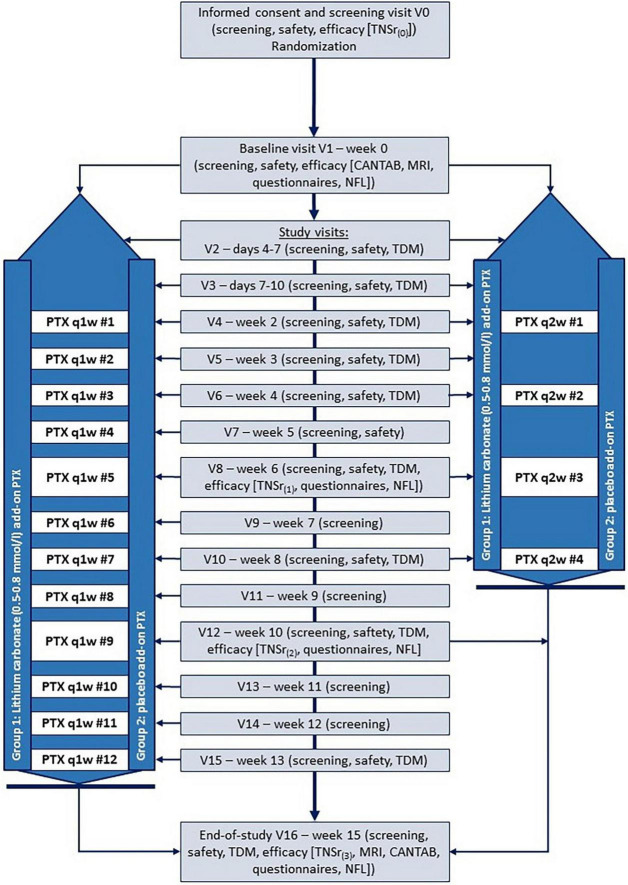
Scheme of intervention in PREPARE trial. CANTAB, Cambridge Neuropsychological Test Automated Battery; MRI, magnetic resonance imaging; NFL, neurofilament light chain protein; PTX, paclitaxel; TDM, therapeutic drug monitoring; TNSr, total neuropathy score reduced.

Participants are treated either with encapsulated lithium carbonate extended release medication (Quilonum^®^ retard) or with encapsulated commercially available placebo tablets. This ensures complete blinding of patients and study physicians. Lithium serum levels are regularly measured according to protocol (Figure 2 and Tables 2, 3) and only reported to two unblinded lithium experts (Charité – Universitätsmedizin Berlin, Dept. of Psychiatry). These lithium experts, who are otherwise not involved in any of the study procedures, enter the data into the electronic case report form (eCRF). For placebo patients, “sham” lithium serum levels are randomly generated and entered into the eCRF to maintain blinding. Dose adjustments are made by study physicians based on reported lithium serum levels in the eCRF and recommendations described in the protocol. This ensures that placebo patients undergo similar dose adjustments as patients on the lithium carbonate medication to maintain proper blinding. This approach has been successfully used in the past in clinical trials evaluating lithium carbonate medication (38).

**TABLE 2:** Overview of study visits and relevant procedures per visit for patients receiving weekly (nab-) paclitaxel infusions (PTX q1w).

Procedure	V0 (screening visit)	V1 (baseline - week 0)	V2 (4–7 days)	V3 (7–10 days)	V4 (week 2 PTX #1)	V5 (week 3 PTX #2)	V6 (week 4 PTX #3)	V7 (week 5 PTX #4)	V8 (week 6 PTX #5)	V9 (week 7 PTX #6)	V10 (week 8 PTX #7)	V11 (week 9 PTX #8)	V12 (week 10 PTX #9)	V13 (week 11 PTX #10)	V14 (week 12 PTX #11)	V15 (week 13 PTX #12)	V16 (week 15)
Chemotherapy																	
Paclitaxel Infusion					X	X	X	X	X	X	X	X	X	X	X	X	
Screening																	
Written informed consent	X																
Inclusion criteria	X																
Exclusion criteria	X																
Discontinuation criteria		X	X	X	X	X	X	X	X		X		X			X	
Demographic data	X																
Medical history	X																
Concomitant therapies/medication	X	X	X	X	X	X	X	X	X	X	X	X	X	X	X	X	X
Karnofsky Index	X	X							X				X				X
Blood ethanol, CDT, Tox. Screen	X																
Tumor characteristics	X																X
SARS-CoV-2 screening #	X	(X)	(X)	(X)	(X)	(X)	(X)	(X)	(X)	(X)	(X)	(X)	(X)	(X)	(X)	(X)	(X)
Safety																	
Adverse events			X	X	X	X	X		X		X		X			X	X
Physical examination	X								X				X				X
Blood pressure/heart frequency	X	X	X	X	X	X	X	X	X		X		X			X	X
Body weight and neck circumference	X	X	X	X	X	X	X		X		X		X			X	X
Body height	X																
ECG ##	X	X			(X)	(X)	X	(X)			X					X	
TDM			X	X	X	X	X		X		X		X			X	X
Safety laboratory	X	X	X	X	X	X	X		X		X		X			X	X
Breast ultrasound in neoadjuvant patients	X																X
Efficacy																	
Neurological examination	X								X				X				X
Electrophysiology	X								X				X				X
Total neuropathy score (TNSr)	X								X				X				X
Neuropsychological testing (CANTAB)		X															X
Brain Imaging (MRI)		X															X
Questionnaires: EORTC-QLQ-C30, EORTC-CIPN20, PHQ-4		X							X				X				X
NFL		X							X				X				X

CANTAB, Cambridge Neuropsychological Test Automated Battery; ECG, electrocardiogram; MRI, magnetic resonance imaging; NFL, neurofilament light chain protein; TDM, therapeutic drug monitoring; TNSr, total neuropathy score reduced.

# Frequency of SARS-CoV-2 screening according to current local regulations; ## additional ECG recordings before and after the paclitaxel infusion for the first 20 patients of the trial at their first 4 infusions.

**TABLE 3:** Overview of study visits and relevant procedures per visit for patients receiving biweekly paclitaxel infusions (PTX q2w).

Procedure	V0 (screening visit)	V1 (baseline – week 0)	V2 (4–7 days)	V3 (7–10 days)	V4 (week 2: PTX #1)	V5 (week 3: TDM)	V6 (week 4: PTX #2)	V8 (week 6 (PTX #3)	V10 (week 8: PTX #4)	V12 (week 10)	V16 (week 15)
Chemotherapy											
Paclitaxel infusion					X		X	X	X		
Screening											
Written informed consent	X										
Inclusion criteria	X										
Exclusion criteria	X										
Discontinuation criteria		X	X	X	X	X	X	X	X		
Demographic data	X										
Medical history	X										
Concomitant therapies/medication	X	X	X	X	X	X	X	X	X	X	X
Karnofsky index	X	X						X		X	X
Blood ethanol, CDT, Tox. screen	X										
Tumor characteristics	X										X
SARS-CoV-2 screening #	X	(X)	(X)	(X)	(X)	(X)	(X)	(X)	(X)	(X)	(X)
Safety											
Adverse events			X	X	X	X	X	X	X	X	X
Physical examination	X							X		X	X
Blood pressure/heart frequency	X	X	X	X	X	X	X	X	X	X	X
Body weight and neck circumference	X	X	X	X	X	X	X	X	X	X	X
Body height	X										
ECG ##	X	X			(X)		X	(X)	X		
TDM			X	X	X	X	X	X	X	X	
Safety laboratory	X	X	X	X	X	X	X	X	X	X	X
Breast ultrasound in neoadjuvant patients	X										X
Efficacy											
Neurological examination	X							X		X	X
Electrophysiology	X							X		X	X
Total neuropathy score (TNSr)	X							X		X	X
Neuropsychological testing (CANTAB)		X									X
Brain MRI		X									X
Questionnaires: EORTC-QLQ-C30, EORTC-CIPN20, PHQ-4		X						X		X	X
NFL		X						X		X	X

CANTAB, Cambridge Neuropsychological Test Automated Battery; ECG, electrocardiogram; MRI, magnetic resonance imaging; NFL, neurofilament light chain protein; TDM, therapeutic drug monitoring; TNSr, total neuropathy score reduced.

# Frequency of SARS-CoV-2 screening according to current local regulations; ## additional ECG recordings before and after the paclitaxel infusion for the first 20 patients of the trial at their first 4 infusions.

A decision whether to unblind a patient is only made after careful evaluation of the patient’s circumstances, symptoms, adverse events and the necessity for emergency medical treatment, where said treatment is based on the knowledge whether the patient received lithium carbonate or placebo to direct further treatment. The need for premature unblinding is evaluated by the study physicians, investigators and with the consultation of the independent lithium experts. In case of a split vote, the DSMB is contacted as final authority. When emergency envelopes are opened, date, time, reason for opening and name of the person opening have to be documented in source data and eCRF. After unblinding the patient has to be immediately excluded from the study.

After completion of the study and data base closure, all participants will be unblinded. Furthermore, the DSMB is granted access to limited randomization lists of subjects with (nab-) paclitaxel treatment in order to recommend trial continuation after interim analysis regarding safety data (cardiac monitoring, maintenance of lithium serum concentrations, renal function, antineoplastic efficacy of (nab-) paclitaxel).

### Intervention

The PREPARE trial is an investigator-initiated proof-of-concept study evaluating co-medication with oral lithium carbonate to prevent (nab-) paclitaxel-induced neurotoxicity. Lithium carbonate is an approved medication, which will be used in an “off-label” indication. The study population consists of female patients with a recent diagnosis of breast cancer who are scheduled to undergo either adjuvant or neoadjuvant chemotherapy with weekly (q1w) (nab-) paclitaxel or biweekly (q2w) paclitaxel infusions. The intervention scheme of our prospective randomized, double-blind, placebo-controlled study approach is summarized in Figure 2. Patients will be recruited at five to eight study sites. The patient’s cancer treatment plan is typically determined in interdisciplinary tumor board meetings. Eligible patients will be informed about the study at their next routine appointment with their primary treating physicians (e.g., gynecologists and oncologists). After written informed consent is obtained, a screening visit V0 is performed, which includes calculation of the primary endpoint TNSr. Eligible patients are then randomly assigned to one of two groups: lithium carbonate add-on to (nab-) paclitaxel (LI + PTX) or placebo add-on to (nab-) paclitaxel (PL + PTX). A baseline visit V1 is scheduled approx. 2 weeks prior to the first (nab-) paclitaxel infusion. At this visit a brain MRI, neuropsychological testing, assessment of patient-reported outcome measures (questionnaires) and safety parameters are recorded. Afterward dosing-in of the trial medication will start gradually increasing the dose every 2–3 days. The first therapeutic drug monitoring (TDM) with measurement of lithium serum concentrations takes place after 4–7 days. While the safety laboratory parameters are visible to all study personnel, the lithium serum levels from TDM are transmitted by the respective laboratories only to two unblinded experts at the Department of Psychiatry, Charité – Universitätsmedizin Berlin. These experts have experience with lithium therapy and potential side effects, are independent as well as spatially separated from the rest of the study personnel, and are not otherwise involved in the trial. They will review lithium serum concentrations and create sham lithium serum levels for placebo patients and transfer TDM data to the eCRF. We expect patients to reach target lithium serum concentrations of 0.5–0.8 mmol/l within the first 10 days by study visit V3, after which patients begin their chemotherapy of weekly (nab-) paclitaxel (PTX q1w) or biweekly paclitaxel (PTX q2w) infusions. During the intervention period of approx. 8 (PTX q2w) respectively, 14 weeks (PTX q1w), patients are initially scheduled for weekly safety visits including TDM (V4–V6), then biweekly (V6–V12) and then monthly (V12 and V16) study visits. The trial medication dose is adjusted by ±1–2 capsules/day depending on the over-/undershoot from the target lithium serum level in both the lithium carbonate and placebo group according to TDM values documented in the eCRF to ensure patient and investigator blinding. Treatment efficacy is assessed at study visits V8, V12, and V16 by calculating the TNSr from patient-reported data, neurological and electrophysiological examination. Secondary efficacy endpoints such as cognitive function, MRI data and supportive laboratory parameters will be assessed at V1 and the last study visit V16. Upon completion of (nab-) paclitaxel, the trial medication is reduced to the evening dose 24 h after the last (nab-) paclitaxel infusion and stopped completely after 96 h. Of note, the intervention period with lithium carbonate is limited to a maximum of 6 paclitaxel infusions for the biweekly and a maximum of 12 paclitaxel infusions for the weekly chemotherapy regimen. After 6 (PTX q2w) respectively 12 (PTX q1w) paclitaxel infusions, lithium carbonate must be reduced to the evening dose 24 h after the last (nab-) paclitaxel infusion and stopped completely after 96 h per protocol. A wash-out period of 2 weeks with no paclitaxel treatment must be guaranteed to assess treatment efficacy of the trial medication lithium carbonate at the final study visit V16 before continuation of paclitaxel chemotherapy. Final analysis of efficacy in the primary efficacy endpoint (TNSr) is done on all randomized patients (intention-to-treat population) at 2 weeks after the last (nab-) PTX infusion (V16 for PTX q1w respectively V12 for PTX q2w). The goal is to show that lithium carbonate is superior to placebo in attenuating CIPN development in addition to demonstrating its safety and feasibility.

#### Frequency and scope of study visits

The number of study visits depends on the patient’s (nab-) paclitaxel treatment regimen. Patients with weekly (nab-) paclitaxel infusions (PTX q1w) will attend 17 study visits, patients with biweekly treatment (PTX q2w) 11 study visits over a total trial period of 16 weeks for each patient. Study visits will be combined with patient’s routine appointments in the gynecological/oncological department, if possible. The study visits focus initially on assessment of eligibility and a baseline neurological evaluation including a clinical neurological and electrophysiological examination of the peripheral nervous system to calculate the TNSr, brain MRI and neuropsychological testing (study visit V0–V1). Study visits subsequent to the start of the trial medication focus on measures for study continuation [‘screening’: discontinuation criteria, assessment of concomitant medication and adverse events, ‘safety’: laboratory parameters including therapeutic drug monitoring (TDM) as well as physical examination and electrocardiogram (ECG)]. Because the trial medication may need dose adjustments, study visits initially take place twice a week (V2 + V3), then weekly (V4 − V6), biweekly (V7 − V10), and finally monthly (V12, V15, and V16). The frequency of study visits exceeds recommendations for treatment with lithium carbonate in patients with mood disorders. After completion of 33% (PTX q1w) respectively, 50% (PTX q2w) as well as 66% (PTX q1w) and 100% (PTX q1w and PTX q2w) of the (nab-) paclitaxel infusions, the primary endpoint TNSr will be assessed (study visits V8, V12, and V16). The final study visit will focus on both neurological and safety endpoints including all the secondary efficacy endpoints (V16) and will conclude the trial. Following advice from the regulatory agency [Bundesinstitut für Arzneimittel und Medizinprodukte (BfArM)], the first 20 patients treated in this trial will undergo intensified cardiovascular monitoring (ECG, heart rate, blood pressure assessment) before and after the first four (nab-) paclitaxel infusions. Additionally tumor response will be analyzed from routine gynecological data (breast ultrasound, TNM stage) in 20 patients with neoadjuvant (nab-) paclitaxel chemotherapy. An interim safety analysis will be conducted after completion of this sub-cohort to ensure that lithium carbonate co-medication is safe and does not interfere with the antineoplastic efficacy of (nab-) paclitaxel. Tables 2, 3 describe the relevant procedures per study visit in detail.

### Outcomes

The primary outcome variable is the Total Neuropathy Score reduced (TNSr), which is a validated score to assess CIPN based on patient-reported symptoms, data from neurological and electrophysiological examination (8). Calculation of the TNSr is performed by assessing seven different variables which are graded with 0 (none) to 4 (pronounced symptoms) points (8). The total score is calculated as sum score at V0, V8, V12, and V16. As secondary outcome variables we will assess patient-reported outcome measures pertaining to self-reported symptoms of CIPN [European Organisation for Research and Treatment of Cancer [EORTC]-CIPN20 (36)], quality of life [EORTC-QLQ-C30 (39)] and symptoms of anxiety and depression [Patient Health Questionnaire (PHQ)-4 (40)]. Additionally, patients’ change in cognitive function will be recorded with the Cambridge Neuropsychological Testing Automated Battery (CANTAB) (41) for the following cognitive domains: processing and psychomotor speed, attention and visual memory, working memory and strategy, verbal learning and memory. Brain MRI imaging data will be assessed including changes in hippocampal volume (42), structural (tractography) (43) and functional connectivity (resting state) (43). As additional clinical endpoints, the number and dose of pain medication for CIPN as well as the cumulative dose of (nab-) paclitaxel that each patient received will be evaluated. In addition, we will analyze blood biomarkers such as serum NFL as a marker for neuroaxonal damage (36). Relevant safety laboratory parameters that are secondary outcome measures are the lithium serum levels from TDM, patients’ serum creatinine and endocrine parameters. Additionally, changes in QTc in ECG recordings will be evaluated as safety parameter as well as the response to (nab-) paclitaxel in neoadjuvantly treated patients determined by breast ultrasound. Table 4 outlines the main outcome variables.

**TABLE 4:** Characteristics and definitions of main endpoints.

Outcome	Instrument	Rating	Domain	Exactly defined outcome	Variable
Primary	TNSr	Clinician rated	Severity of CIPN	Mean change from baseline	Continuous
Secondary/efficacy	EORTC-QLQ-C30	Self-rated	Quality of life	Median change from baseline	Continuous
Secondary/efficacy	EORTC-CIPN20	Self-rated	Symptoms of CIPN and severity	Median change from baseline	Continuous
Secondary/efficacy	PHQ4	Self-rated	Symptoms of anxiety/depression	Median change from baseline	Continuous
Secondary/efficacy	Serum NFL	Laboratory value	Severity of neuroaxonal damage	Mean change from baseline	Continuous
Secondary/efficacy	CANTAB	Calculated	Severity of cognitive impairment	Median change from baseline	Continuous
Secondary/efficacy	MRI	Expert clinician rated/Calculated	Brain function	Mean change from baseline	Continuous
Secondary/efficacy	Comedication	N/A	Severity of pain	Median change from baseline	Continuous
Secondary/efficacy	Paclitaxel dose	N/A	Tolerance to chemotherapy	Median cumulative dose	Continuous
Secondary/safety	Lithium serum levels	Laboratory value	Pharmacovigilance	Mean concentration. Measurements outside target concentration.	Continuous. Ordinal.
Secondary/safety	Serum creatinine	Laboratory value	Metabolism	Mean concentration	Continuous
Secondary/safety	eGFR	Laboratory value	Metabolism	Mean change from baseline	Continuous
Secondary/safety	TSH, fT3, fT4	Laboratory values	Metabolism	Mean change from baseline	Continuous
Secondary/exploratory safety	Breast ultrasound	Clinician rated	Tumor response	Median change from baseline	Continuous
Secondary/safety	QTc in ECG	N/A	Safety	Mean change from baseline	Continuous
Secondary/safety	AE, SAE	Clinician rated	Safety	Number. Grade.	Continuous. Ordinal.

### Permitted co-medications/co-treatments

Preclinical studies have demonstrated a pharmacodynamic interaction of lithium carbonate with paclitaxel in terms of a protective effect in neuronal cells and cardiac myocytes, and therefore lithium carbonate is now being tested in the PREPARE trial to prevent paclitaxel-induced neurotoxicity. As most patients receive a complex chemotherapy regimen, other chemotherapeutic drugs that are part of current state of the art routine standard care for breast cancer set by current gynecological/oncological guidelines are permitted during the trial. For most patients this includes a combination chemotherapy with epirubicin or doxorubicin and cyclophosphamide. HER2neu positive patients additionally receive HER2 targeting antibodies (trastuzumab and pertuzumab) in parallel to (nab-) paclitaxel treatment, which are also permitted concomitant medications during the trial. Equally permitted are routine premedication of (nab-) paclitaxel to limit its side effects. Furthermore, filgrastim or pegfilgrastimare are permitted to prevent neutropenia. A smaller percentage of patients (triple negative, estrogen receptor negative and HER2neu positive) may require combination therapy of (nab-) paclitaxel with carboplatin, which is also neurotoxic to a lesser extent. In our own cohort of ovarian and breast cancer patients, a combination of (nab-) paclitaxel and carboplatin was, however, not more neurotoxic than (nab-) paclitaxel chemotherapy alone (36). Therefore, these patients will be enrolled in the PREPARE trial as the primary endpoint is not expected to be compromised. Our literature research in several open access databases (PubMed, Clinicaltrials.gov, Cochrane library etc.), but also the GlobalData database also did not reveal any increased safety risks for the combination of lithium carbonate and carboplatin. Antihormonal substances are an established part of routine standard care for breast cancer and also permitted in this trial.

Co-medication which are known to cause increased neurotoxicity due to increased lithium serum levels are not permitted (44). These include phenytoin, fosphenytoin, carbamazepine, methyldopa and tricyclic antidepressants. In case of co-medications, which can potentially interfere with lithium carbonate – especially diuretics, certain antibiotics (ampicillin, tetracyclines, aminogly-cosides, and metronidazole), non-steroid anti-inflammatory drugs (NSAID), ACE and angiotensin II receptor antagonists (44) – initially additional weekly study visits need to be scheduled to assess kidney function and lithium levels. For some co-medication, in addition to weekly TDM dose adjustments to lower target lithium serum levels are advised, these include calcium antagonists and calcium channel blockers.

Equally permitted are drugs for treating neuropathic pain such as gabapentin, pregabalin and duloxetine. In our clinical experience, treatment with these drugs usually decreases the intensity of sensory symptoms, but does not alter the localization (which is more relevant for calculating the TNSr sum score) and does not impair objective measurements. Thereby interference with the primary endpoint is not expected. Frequency and dose of these pain medications are assessed as an additional secondary endpoint.

Many gynecologic and oncologic treatment facilities use voluntary cooling of the extremities as part of their routine standard care for breast cancer treatment even though demonstration of a clear clinical benefit is still lacking. In order to obtain data which is relevant to the real world situation, voluntary cooling of the extremities as part of routine standard care in breast cancer treatment is permitted during the intervention. All study participants are offered a standardized set of coolpacks to use for the cooling process and the method of cooling, number of extremities and number of (nab-) paclitaxel cycles with cooling will be recorded.

### Safety

All breast cancer patients enrolled in the trial will be treated according to current standard of care guidelines set by the German consortium of gynecological oncology [Arbeitsgemeinschaft Gynäkologische Onkologie (AGO)]. Both lithium carbonate and paclitaxel have long been approved drugs for various indications and have a proven safety profile. As lithium carbonate is eliminated solely via the kidneys and paclitaxel is metabolized in the liver, no pharmacological interactions are to be expected. Building on experience for lithium carbonate therapy in psychiatric patients, we will educate patients about the safety profile of the trial medication and closely monitor the patients’ safety. Owing to the increased risk for alterations in fluid balance in chemotherapy patients, our proposed schedule of trial visits (Figure 2) surpasses clinical recommendations for use of lithium carbonate and guarantees that any unwanted additional side effects from the trial medication are rapidly identified. All adverse events (AE) reported by the patient or observed by the investigators will be documented and assessed to ensure a sufficient surveillance on the safety of the patients. All (serious) AEs and suspected unexpected serious adverse reaction (SUSARs) will be treated appropriately. Such treatment may include changes in trial medication treatment including possible interruption or discontinuation, starting or stopping concomitant treatments, changes in the frequency or nature of assessments, hospitalization or any other medically required intervention. Standard procedures for reporting of (S)AEs and SUSARs are used according to legislation and standards, Standard Operating Procedures (SOPs) and the quality management protocol of the Charité Clinical Trials Office. As required, the competent authorities, the ethics committees, the investigators and the DSMB will be informed on all safety issues, which could be a risk for all patients or change the risk-benefit value of the study as well as on all reportable single events (e.g., SUSARs). Definition of all safety information will be used as defined by applicable law/guidelines, study centers’ SOPs and the protocol.

Patients who need to prematurely terminate (nab-) paclitaxel due to side effects will discontinue the trial medication with lithium carbonate/placebo by reducing to the evening dose 24 h after the last (nab-) paclitaxel infusion and stopping completely after 96 h. Patients who terminate (nab-) paclitaxel prematurely must continue with regular study visits assessing primary and secondary outcome measures at V8, V12, and V16 to avoid negative selection bias.

An early discontinuation of the trial may be decided if new scientific data during the course of the trial changes the risk-benefit-balance significantly. If such data emerges, recruitment and treatment of currently treated patients will be paused immediately. Due to the low number of participants enrolled, there is no formal interim analysis regarding efficacy planned. An interim safety analysis will be performed after the completion of 20 patients with (nab-) paclitaxel treatment. The DSMB will review the data and analysis and formulate a recommendation if the trial should continue. The trial will be prematurely terminated if there is an increased number of SAE in the lithium carbonate group, especially if (1) lithium serum concentrations (asymptomatic or symptomatic) of >1.5 mmol/l are observed in >20% of patients (evaluation by unblinded independent lithium experts), (2) rates of severe cardiac arrhythmia exceeds >20% of patients in the lithium carbonate group, (3) increased rate of renal disease with eGFR <60 ml/min/1.73 m^2^ is observed in >20% of patients in the lithium carbonate group. To ensure patient safety, regular (every 6 months) and close oversight is executed by the DSMB. The whole trial will be stopped upon request of the DSMB if severe safety concerns related to the intervention should arise. The trial will also be terminated if the ethics committee and/or regulatory agency withdraws the approval of the study.

### Data management

Patient related data will be recorded in study files (source data) under the participant’s real name. The information required by the protocol will be transferred electronically to a central database at Charité – Universitätsmedizin Berlin by means of an electronic case report form (eCRF – secuTrial, interactive Systems Berlin, Germany) under a pseudonym. Every patient at each study site will receive a pseudonym unique for this individual patient. All patient identifying data remain at the individual study site. All data required for analysis are acquired at each study site and transferred electronically via the eCRF to the coordinating center (Charité – Universitätsmedizin Berlin, Charité Clinical Trials Office). Daily backups are performed on the remote servers used to digitally store all trial data. The Charité Clinical Trials Office data management team and the coordinating investigators will perform frequent consistency and plausibility checks on all entered data in the eCRF. All source data will be monitored by a study-independent monitor from the NeuroCure Clinical Research Center. Afterward, the database will be closed and the data matrix transferred to the trial statistician in pseudonymized form for statistical analysis.

### Data analysis plan

The final analysis will be conducted on all randomized patients in the intention-to-treat population.

#### Primary analysis

Confirmatory analysis will be conducted based on the intention-to-treat (ITT) population, defined on the basis of the ITT principle. The aim is to show that the lithium carbonate group is superior to the placebo control group, meaning that the mean TNSr at 2 weeks after the last (nab-) paclitaxel infusion adjusted for the baseline value is lower in the lithium carbonate group than in the placebo control group. An analysis of covariance (ANCOVA) adjusted for baseline TNSr values using group allocation, study center and combination treatment of paclitaxel with carboplatin as factors will be applied (45). The global two-sided significance level is 0.1. Missing data will be categorized according to Rubins system of “missing completely at random,” “missing at random,” and “missing not at random” (MCAR, MAR, and MNAR) (46). For missing data in outcome variables, data will be imputed using multiple imputation-chained equations (mice) (47), where appropriate with the underlying data structure assumptions. It is planned to perform sensitivity analyses, in which the robustness of the inference will be investigated.

#### Secondary analyses

A sensitivity analysis to the primary efficacy ANCOVA model will be applied with additional covariates/factors given by age, body mass index, progesterone receptor status and chemotherapy regimen and cumulative dose (48). Descriptive methods will be used for analysis of all clinical, demographic and safety parameters, including the calculation of appropriate summary measures of the empirical distribution as well as 95% confidence intervals and calculation of descriptive two-sided *p*-values. Additionally, sensitivity analyses will be conducted for different populations (per-protocol population).

### Patient and public involvement

Our group is actively involved in patient education activities of the Charité Comprehensive Cancer Center, the Stiftung Eierstockkrebs, Nord-Ostdeutsche Gesellschaft für Gynäkologische Onkologie e.V. (NOGGO), the Charité Breast Cancer self-help group, the BRCA Netzwerk e.V., and Frauenselbsthilfe nach Krebs e.V. self-help groups. These activities underscored the high medical need and provided much needed feedback and motivation for the design of the proposed study and protocol. Representatives of different patient self-help organizations (Frauenselbsthilfe nach Krebs e.V., BRCA Netzwerk e.V., and Charité Breast Cancer self-help group) inspired the trial and gave input to the protocol to include patients’ needs and to define relevant endpoints.

## Discussion

Here we present the protocol of a multicenter, randomized, double-blind, placebo-controlled trial to prevent paclitaxel-induced neurotoxicity by co-medication with lithium carbonate. According to preclinical data obtained in different laboratories across the world in different preclinical models involving different species and considering limited data from a retrospective clinical analysis, a beneficial effect of lithium carbonate with regard to the prevention of paclitaxel-induced polyneuropathy and cognitive impairment is to be expected. Although an oral treatment regimen with lithium carbonate requires a longer treatment period of 8–14 weeks and increased clinical and paraclinical monitoring, it is also the most reliable way to ensure target lithium serum concentrations of 0.5–0.8 mmol/l.

Potential adverse effects could result from the combination treatment of lithium carbonate with paclitaxel, although they are pharmacologically unlikely and have not been observed in preclinical experiments to date (16, 21, 49). These include a potential increase in cardiac arrhythmias and increased serum lithium concentrations due to fluid imbalance in patients undergoing paclitaxel chemotherapy. To detect potential adverse effects as early as possible and to ensure patient safety, intensive cardiac monitoring before and after the first four paclitaxel infusions is mandatory in the first 20 randomized patients with recordings of vital signs and additional ECG. Safety data (cardiac monitoring, maintenance of lithium serum levels, time to reach intended lithium serum levels, renal function) from these first 20 randomized patients will be evaluated by the DSMB in an interim safety analysis. Major fluid imbalance (e.g., due to increased vomiting or diarrhea) in paclitaxel-treated patients that could lead to increased or even toxic lithium serum levels is highly unlikely, as only a very small proportion of patients (<5%) experience these symptoms during paclitaxel treatment (50). Another possible unintended effect could be a decreased cytotoxic capacity of paclitaxel with concomitant administration of lithium carbonate, although data from tumor-transplant models do not suggest such findings. We will analyze tumor response rates using ultrasound and pathology data in all patients receiving neoadjuvant paclitaxel therapy. Potential study pitfalls arise from the need for repeated measurements of serum lithium concentrations to guide treatment while the study personnel themselves remain blinded. We used a previously established method with two unblinded independent reviewers of these laboratory data obtained at all trial sites and the generation of sham concentrations in placebo-treated patients to achieve comparable dose adjustments in the lithium carbonate and placebo group. Detailed laboratory procedures, extensive training of study personnel, and SOPs ensure adherence to the protocol and workflow to counteract accidental unblinding.

In terms of benefits for the patients, a positive result of the PREPARE trial – even if only applicable to patients receiving (nab-) paclitaxel therapy – would constitute a medical breakthrough. It would increase cancer patients’ chances of survival and substantially improve their quality of life in the short and long term. (Nab-) paclitaxel is a first line treatment for many solid tumors worldwide. Thereby, the potential clinical impact of the proposed trial is highly significant. As many cancer patients keep working or aspire to return to their occupation after the treatment is finished, neurological sequelae after chemotherapy such as CIPN and PCCI not only have a profound individual, but also a very significant socioeconomic impact. Prevention of (long-term) adverse conditions both in the peripheral and central nervous system would save money on costly and often ineffective drug therapy, physiotherapy, or cognitive training, as well as help to reintegrate patients faster into work life.

In conclusion, though a combination treatment of lithium carbonate and paclitaxel has not been systematically studied in a prospective design and some safety concerns arise when treating (severely) diseased cancer patients, it needs to be stressed, that both drugs (lithium carbonate and paclitaxel) are extremely well characterized and have market authorization, which is why the combination is already possible for instance in psychiatric patients (22). Furthermore it has to be considered, that the benefits for the patients – in case of a reasonable positive result – outweigh potential negative effects. Breast cancer patients are an ideal cohort for an exploratory trial such as this as they are overall relatively healthy compared to other cancer patients regarding comorbidities, other medication, risk factors etc. In addition, mostly patients with early breast cancer will be included in the PREPARE trial which further takes safety concerns of such a complex intervention into consideration.

Lastly, a positive result of the PREPARE trial could inform a larger confirmatory phase-3 trial including patients of both genders and different tumor entities with the aim to initiate a guideline change. As chemotherapy treatment remains to be a substantial part of antineoplastic treatment in the foreseeable future, there is an urgent need for the development of neuroprotective strategies in chemotherapy treatment.

## Ethics and dissemination

This trial will be conducted in accordance with the current ICH-GCP-guidelines. Good Clinical Practice (GCP) is an international ethical and scientific quality standard for designing, conducting, re-cording and reporting trials that involve the participation of human subjects. Compliance with this standard provides public assurance that the rights, safety and well-being of trial subjects are protected, consistent with the principles that have their origin in the Declaration of Helsinki, and that the clinical trial data are credible. Informed consent will be obtained from all study participants. The study protocol, patient information and consent form have been approved by the state ethics committees and the federal authority (BfArM). The leading Ethics Committee will immediately be informed (by the sponsor/sponsor representative) of all changes to the protocol (according to GCP-V §10) and of all events that could affect a patients’ safety. The Ethics Committee and competent authority will also be informed of all suspected SUSARs and of regular or premature termination of the study. The trial was preregistered in the German Clinical Trials Register (DRKS00027165). The study results will be published irrespective of the study outcome in peer-reviewed journals and at (inter-)national conferences, preferably applying an open access and open data publication strategy.

### Study management

The overall responsibility for the study lies with the sponsor/sponsor representative Charité – Universitätsmedizin Berlin. The principle investigator Matthias Endres together with each investigator of the other study sites is clinically responsible for the research team at each study site. There will be close oversight by the DSMB, which consists of an expert in lithium therapy, an expert in clinical trials and statistics and an expert in breast cancer treatment. The DSMB charter is available upon reasonable request from the corresponding author.

A regulatory authority or representatives of an ethics committee can visit the study centers for inspections. Additionally, the sponsor will appoint authorized representatives to monitor the study conduction at each study site. Audits and inspections may be performed to independently evaluate all study related procedures to determine whether these were conducted according to the protocol, GCP, ICH guidelines and any potential regulatory requirements including data acquisition, recording, analysis and reporting.

### Ethics approval

The study has been approved by the appropriate ethics committee (Ethik-Kommission des Landes Berlin and Landesamt für Gesundheit und Soziales Berlin) under reference number 21/232 – IV E 10 on January 11, 2022.

## Author contributions

ME was the principle investigator of the study. PH and WB were coordinating investigators and wrote the manuscript. SL and SM led the administrative office responsible for the study. PH, WB, ME, and NB designed the study. PH, WB, and ME acquired funding for the study. PH, WB, SL, SM, FP, and TS-H wrote the study protocol and investigator’s brochure and obtained approval from the ethics committee and regulatory agency. CE wrote the investigator medicinal product dossier (IMPD) and was responsible for the production and distribution of the trial medication. GR provided the sample size calculation and statistical analysis plan. NB, AF, SA, PS, IM-H, OH, and TZ are leading investigators at the external trial sites. All authors reviewed the study protocol and reviewed the manuscript.
